# Zika Virus–Related News Coverage and Online Behavior, United States, Guatemala, and Brazil

**DOI:** 10.3201/eid2207.160415

**Published:** 2016-07

**Authors:** Brian G. Southwell, Suzanne Dolina, Karla Jimenez-Magdaleno, Linda B. Squiers, Bridget J. Kelly

**Affiliations:** RTI International, Research Triangle Park, North Carolina, USA

**Keywords:** Zika virus, flavivirus, mass media, Internet, social media, Twitter, messaging, communications, media, viruses, United States, Guatemala, Brazil

**To the Editor:** News coverage of emerging infectious diseases tends to be episodic and ephemeral rather than thematic, comprehensive, and consistent over time, in part because of newsroom constraints (*1**–**3*). Public health authority announcements may help drive peaks in coverage and warrant attention, in particular given the importance of trust and credibility for information acceptance (*4**,**5*). Moreover, online search behavior and social media interaction tend to respond to news coverage, especially for novel health issues (*6**,**7*).

The nature of Zika virus transmission as a novel phenomenon not completely understood by researchers could encourage anxiety and fear among the public (*8**,**9*). Patterns of social interaction and search behavior regarding Zika virus can point to opportunities and constraints for education efforts.

To assess relationships between news coverage, social media mentions, and online search behavior regarding Zika virus, we studied data available for January 1–February 29, 2016. Although news outlets occasionally covered Zika virus before 2016, our selected period included prominent announcements. For example, on January 28, the World Health Organization declared that Zika virus was “spreading explosively” (*10*), and the Centers for Disease Control and Prevention issued a travel alert. On February 3, authorities reported the first case that appeared in the United States.

Across 3 data sources, we searched for mentions of “Zika” or “El Zika.” We used Google Trends (Google Inc., Mountain View, CA, USA) to assess the number of total searches that originated in the United States, Guatemala, or Brazil for these terms, relative to total Google searches for any topic for the same period. We used a scale of 0–100 (as an indicator of relative volume), with 50 representing half the volume as 100 but not a specific absolute number. Zika virus has been detected in >25 countries since 2015; the countries selected were places where transmission has been relatively widespread or where Zika virus had not yet been but was anticipated to be. We used a monitoring tool, Crimson Hexagon (http://www.crimsonhexagon.com/), to capture the total number of daily Twitter posts (tweets) and focused on tweets geotagged as originating from the United States, Guatemala, or Brazil. Last, we counted Associated Press news wire stories as a proxy for daily volume of Zika news coverage in the Western Hemisphere (Technical Appendix).

Using a day as our unit of analysis (i.e., n = 60 in the analysis), we first assessed Pearson product-moment correlations between news coverage, social media mentions, and online search behavior and then fit a time series model. Results suggested prominent but ephemeral peaks in salience and attention, with some variation over time in searches by country (Figure). We found strong positive correlations between news (daily volume) and tweets for all 3 countries (United States, *r* = 0.86, p<0.001; Guatemala, *r* = 0.78, p<0.001; Brazil, *r* = 0.60, p<0.001). We also found strong positive correlations between news and Google searches for all 3 countries (United States, *r* = 0.86, p<0.001; Guatemala, *r* = 0.74, p<0.001; Brazil, *r* = 0.48, p<0.001). Because time series data can reflect autocorrelation that makes observed relationships spurious, interpretation of bivariate correlations alone to link time series data is inadvisable. To assess the relationship between news coverage and online searching related to Zika virus, we used time series analysis to predict US Google searches as a function of other observed trends and date. We fit an autoregressive integrated moving average (0, 1, 3) model to address dependence between residuals, resulting in a Ljung-Box statistic that was not significant (p>0.05). This finding indicated that we sufficiently reduced the time series to white noise to assume no autocorrelation in residuals. Our model achieved an R^2^ value of 0.90 and stationary R^2^ value of 0.53. Associated Press wire stories emerged as a significant and positive predictor (coefficient = 1.52, *t* = 3.24, p<0.01). No other predictor predicted variance greater than that of news stories (p>0.05). Daily news story volume predicted departures from the expected trend in US search behavior related to Zika virus.

**Figure:**
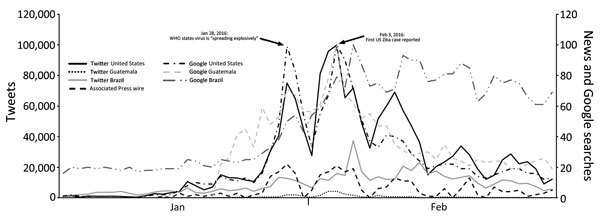
Comparison of number of tweets by individual persons, Google searches by individual persons, and Associated Press news stories about Zika virus in the United States, Guatemala, and Brazil, January 1–February 29, 2016.

Our results suggest that news coverage of public health authority announcements opens brief windows of information sharing, engagement, and searching that offer opportunities to address perceptions and provide preparation and vector control recommendations through education. Sharing and searching are less apparent outside these windows, especially in contexts in which an emerging infectious disease is not yet prevalent. Our findings may not generalize beyond the initial stages of Zika virus transmission in the United States, and future work could obtain appropriate data for investigating the tone of news coverage and online communication in various countries. Nevertheless, recent trends in online information-seeking about Zika virus has been sensitive to official announcements, suggesting the usefulness of pairing announcements with provision of information resources that can be found through search engines.

Technical Appendix Detailed methods for analysis of Zika virus–related news coverage and online behavior and summary of data used for the analysis.
