# Supplementary material for: Zika Virus–Related News Coverage and Online Behavior, United States, Guatemala, and Brazil
Source: Emerg Infect Dis. 2016 Jul;22(7):1320–1. doi: 10.3201/eid2207.160415 (PMC4918164; doi:10.3201/eid2207.160415)
Supplement: Technical Appendix — Detailed methods for analysis of Zika virus–related news coverage and online behavior and summary of data used for the analysis. [file 16-0415-Techapp-s1.pdf]

Article DOI: http://dx.doi.org/10.3201/eid2207.160415

Zika Virus–Related News Coverage and Online Behavior, United States, Guatemala, and Brazil

Technical Appendix

Detailed Methods

Data collection

In addition to using search score data available from Google, we used 2 tools to capture news coverage and social media mentions. We used a licensed tool called Meltwater (San Francisco, CA, USA) to capture Associated Press news stories during January 1–February 29, 2016, although researchers without a Meltwater license also could capture stories manually from the Associated Press wire each day with the appropriate subscription to the wire. For Twitter mentions, we purchased a license to the full census of all Twitter posts in the countries tracked through a tool offered by Crimson Hexagon (Boston, MA, USA). Our use of a full census of Twitter mentions represents a departure from other methods that offer only a sample. We purchased access to the total count of all Twitter mentions and used the search terms described in the paper. Although the array of tools we used may not be freely available to all researchers, we include the data used in our analysis for each news or social media outlet we examined (Technical Appendix Table).

Analysis

Data analysis that assesses relationships between longitudinal variables or data organized by time should acknowledge the possibility of autocorrelation in a time series that can violate assumptions of independence between observations (*1*). For the time series analysis presented, we fit an autoregressive integrated moving average (ARIMA) (0, 1, 3) model by using IBM SPSS Statistics 21 (Armonk, NY, USA). We opted to test this model after inspecting the original time series of dependent variable values by using the partial autocorrelation function and the autocorrelation function to assess whether autoregressive or moving average components would

be useful in eliminating autocorrelation in the time series. Because the partial autocorrelation function declined after 1 lag and the autocorrelation function declined after 3 lags, we fit an ARIMA (0, 1, 3) model, which resulted in a series without apparent autocorrelation in the residuals: the resulting Ljung-Box statistic (23.60, DF = 15) was not significant ($p > 0.05$), indicating that we did not need to reject a hypothesis of no autocorrelation in the residuals. In other words, we applied an ARIMA model to enable our independent variables to predict a prewhitened series that accounted for potential autocorrelation in the dependent series. Specifically, we fit a model to predict a US Google search score that included 4 elements that reflected the dependent variable time series (a difference parameter and 3 moving average parameters) and a set of 7 predictors: a day variable along with Associated Press wire story counts, US Twitter mentions, Guatemala Twitter mentions, Brazil Twitter mentions, a Guatemala search score, and a Brazil search score. Stationary R-squared value for the model was 0.53. Root mean squared error was 8.8.

Reference

1. McCleary R, Hay RA, Meidinger EE, McDowall D. Applied time series analysis for the social sciences. Thousand Oaks (CA): Sage Publications; 1980.

Technical Appendix Table. News coverage and social media mentions of Zika virus by various media channels and day, January 1–February 29, 2016

Month/day	Associated Press wire	Google United States	Google Guatemala	Google Brazil	Twitter United States	Twitter Guatemala	Twitter Brazil
1/1	0	1	0	16	1,059	2	1,393
1/2	0	2	0	20	1,295	10	1,736
1/3	0	1	0	19	414	2	1,979
1/4	0	1	0	20	1,137	10	2,459
1/5	0	1	0	19	1,039	12	3,556
1/6	0	1	0	20	990	8	3,587
1/7	0	1	0	19	1,076	9	3,605
1/8	0	1	0	18	887	7	4,212
1/9	0	0	0	20	583	2	2,883
1/10	0	0	0	17	465	5	1,850
1/11	0	1	0	18	1,385	12	2,357
1/12	0	2	0	18	2,725	20	3,760
1/13	1	2	0	19	2,405	39	4,460
1/14	0	2	0	19	3,310	0	4,476
1/15	3	5	0	19	3,907	8	3,510
1/16	1	8	0	25	10,602	47	6,458
1/17	2	10	0	24	7,844	22	3,359
1/18	1	9	0	21	5,251	75	3,059
1/19	2	9	0	20	6,931	31	4,167
1/20	3	14	22	25	15,044	200	5,884
1/21	1	12	26	24	13,194	159	4,686
1/22	3	11	41	23	12,960	423	5,160
1/23	0	13	45	25	11,664	434	6,340
1/24	2	10	33	25	10,682	262	4,300
1/25	3	16	59	29	17,191	582	6,086
1/26	14	30	48	33	30,654	499	7,353
1/27	17	48	53	38	40,411	704	13,386

Month/day	Associated Press wire	Google United States	Google Guatemala	Google Brazil	Twitter United States	Twitter Guatemala	Twitter Brazil
1/28	22	99	71	50	75,014	1,882	12,663
1/29	16	83	67	54	64,885	1,987	11,331
1/30	0	54	57	43	44,834	1,020	9,006
1/31	5	33	56	54	27,524	429	6,555
2/1	15	55	61	59	81,131	1,626	12,525
2/2	17	67	78	68	95,771	4,214	11,436
2/3	21	100	100	79	99,830	4,291	15,895
2/4/	17	90	68	72	65,482	2,320	14,576
2/5	19	71	62	100	71,777	1,693	37,342
2/6	5	51	52	87	49,016	729	17,195
2/7	0	37	55	73	32,508	396	11,443
2/8	6	33	47	75	51,640	1,554	13,747
2/9	7	41	47	69	59,935	796	12,009
2/10	13	40	35	71	69,056	847	12,243
2/11	11	37	31	93	57,113	678	20,615
2/12	11	29	28	90	47,183	846	17,785
2/13	3	24	29	90	36,202	183	23,147
2/14	1	19	30	76	14,995	70	15,587
2/15	1	21	25	77	20,468	358	17,383
2/16	6	22	35	81	23,404	505	14,014
2/17	0	22	26	81	27,197	509	12,314
2/18	6	19	24	88	33,825	436	13,391
2/19	13	19	21	85	28,167	461	14,468
2/20	2	14	25	63	18,913	148	9,629
2/21	0	13	23	68	12,041	41	6,376
2/22	7	15	24	80	14,798	203	9,026
2/23	4	17	24	75	24,839	239	11,683
2/24	4	19	19	77	28,650	203	11,103
2/25	5	19	19	75	22,237	271	9,179
2/26	1	16	22	66	23,589	246	9,465
2/27	2	16	24	61	18,998	83	7,436
2/28	3	12	26	61	9,083	63	4,649
2/29	5	13	19	69	12,162	41	5,670
